# Recommendations for the management of myasthenia gravis in Belgium

**DOI:** 10.1007/s13760-024-02552-7

**Published:** 2024-04-22

**Authors:** Jan L. De Bleecker, Gauthier Remiche, Alicia Alonso-Jiménez, Vinciane Van Parys, Véronique Bissay, Stéphanie Delstanche, Kristl G. Claeys

**Affiliations:** 1 Department of Neurology, University Hospital Ghent and AZ Sint-Lucas, Ghent, Belgium; 2 Centre de Référence Neuromusculaire, Department of Neurology, Hôpital Universitaire de Bruxelles (HUB)-Hôpital Erasme, Université Libre de Bruxelles (ULB), Brussels, Belgium; 3 Department of Neurology, Faculty of Medicine and Health Sciences, Antwerp University Hospital, Translational Neurosciences, UAntwerpen, Antwerp, Belgium; 4 Department of Neurology, Cliniques Universitaires Saint-Luc, Université Catholique de Louvain (UCL), Brussels, Belgium; 5 Vrije Universiteit Brussel, Universitair Ziekenhuis Brussel, NEUR Research Group and Department of Neurology, Brussels, Belgium; 6 University Department of Neurology, CHR Citadelle, Liège, Belgium; 7 Department of Neurology, University Hospitals Leuven, Leuven, Belgium; 8 Laboratory for Muscle Diseases and Neuropathies, Department of Neurosciences, KULeuven, and Leuven Brain Institute (LBI), Leuven, Belgium

**Keywords:** Myasthenia gravis, Clinical management, Treatment recommendations, Therapies, Belgium

## Abstract

International guidelines on the treatment of myasthenia gravis (MG) have been published but are not tailored to the Belgian situation. This publication presents recommendations from a group of Belgian MG experts for the practical management of MG in Belgium. It includes recommendations for treatment of adult patients with generalized myasthenia gravis (gMG) or ocular myasthenia gravis (oMG). Depending on the MG-related antibody a treatment sequence is suggested with therapies that can be added on if the treatment goal is not achieved. Selection of treatments was based on the level of evidence of efficacy, registration and reimbursement status in Belgium, common daily practice and the personal views and experiences of the authors. The paper reflects the situation in February 2024. In addition to the treatment considerations, other relevant aspects in the management of MG are addressed, including comorbidities, drugs aggravating disease symptoms, pregnancy, and vaccination. As many new treatments might potentially come to market, a realistic future perspective on the impact of these treatments on clinical practice is given. In conclusion, these recommendations intend to be a guide for neurologists treating patients with MG in Belgium.

**Supplementary Information:**

The online version contains supplementary material available at 10.1007/s13760-024-02552-7.

## Introduction

Myasthenia gravis (MG) is a chronic autoimmune disease of the neuromuscular junction, which can cause debilitating and potentially life-threatening muscle weakness [1]. In adults, MG is a rare disease where most neurologists might encounter a new MG patient only once every 2 years [2]. MG is estimated to affect more than 700,000 people globally, with European incidence ranging between 0.63 and 2.9 per 100,000 person-years and prevalence ranging between 11.2 and 36.1 per 100,000 persons [3]. In children, MG is even more rare, with European incidence rates ranging between 0.09 and 0.43 per 100,00 person-years [4]. There is a peak in incidence from 60 to 70 years for both genders and an additional peak from 20 to 40 years in women [5]. An overall increase in prevalence has been observed over the past decades, mostly due to better diagnosis and treatment, and increased longevity of the overall population [2].

Clinically, MG can be mainly recognized by its fatigable and fluctuating muscle weakness. Ocular weakness is the initial symptom in 70–75% of MG patients, resulting in ptosis and/or diplopia [5]. Up to 80% of patients with ocular symptoms subsequently develop generalized MG (gMG) within 2 years. In patients with gMG the ocular, facial, bulbar, respiratory and limb muscles can be affected, causing reduced facial expression, dysphagia, dysarthria, dyspnoea, and/or (proximal) limb weakness [6]. Symptom severity may vary, with patients reporting no symptoms to patients with heavily affected quality of life (QoL) [7] due to weakness, fatigue, a lack of physical energy [8], and poor sleep quality [9]. MG considerably impacts patients’ ability to work and perform daily activities, with more severe disease associated with greater impact [10]. If symptoms are not controlled properly, exacerbations can result in life-threatening myasthenic crises characterized by respiratory insufficiency or even death [11].

MG is caused by autoantibodies resulting in defective signal transduction at the neuromuscular junction or endplate [6]. Pathogenic immunoglobulin G (IgG) autoantibodies target acetylcholine receptors (AChRs) and other structural components of the neuromuscular junction—muscle-specific kinase (MuSK) and lipoprotein receptor-related protein-4 (LRP4)—impairing neuromuscular transmission and leading to muscle weakness and fatigability [12]. Anti-AChR antibodies are detected in around 85% of patients with gMG [1] and 50% with ocular MG (oMG) [5]. The remaining 15% of gMG patients have MuSK autoantibodies (5%), LRP4 autoantibodies (1–3%), or no detectable antibodies (seronegative MG) (10–15%) [1, 12]. The thymus is involved in many MG patients. In AChR positive (AChR +) gMG patients, 10% present with thymoma, an often relatively benign tumour of the thymus, and up to 70% of early onset (< 50 years) gMG patients presents with thymus hyperplasia [13, 14].

Although no curative treatment is currently available for MG, the disease can be reasonably controlled in most patients. A recent study from a Belgian neuromuscular reference centre (NMRC) demonstrated that—when proper treatment is initiated early—up to half of the patients can live symptom free, and half of the symptomatic patients experience only mild symptoms allowing normal activities of daily living (ADL) [5]. Because MG is heterogeneous, no one treatment approach is best for all patients. Few physicians treat enough patients with MG to be comfortable with all available treatments [15]. Therefore, consensus international guidelines have been developed to guide clinicians on the multifaceted approach to manage MG [15, 16]. These international guidelines may not always be applicable to the Belgian situation since they do not consider the Belgian registration and reimbursement status of MG treatments or common clinical practice in Belgium.

The current publication provides recommendations for the treatment of adult MG in Belgium in February 2024. The recommendations are based on the level of evidence, registration and reimbursement status of MG treatments in Belgium in February 2024, common daily practice and the personal views and experiences of the authors.

## Methodology

The recommendations for treatment of MG presented in this paper were prepared in February 2024 by a group of Belgian MG experts. Starting point for the discussion were international published MG guidelines (Myasthenia Gravis Foundation of America [15, 16], Spierziekten centrum Nederland [17], Deutsche Gesellschaft für Neurologie [18, 19], and the Association of British Neurologists [20, 21]). Additionally, a literature search was performed to identify relevant academic publications and MG clinical trials published after the cut-off dates used in the international guidelines. The recommendations from the published guidelines and the relevant publications identified in the literature search were evaluated for their applicability to the Belgian situation (registration status, reimbursement, clinical practice) during several experts meeting with one representative of each of the seven Belgian NMRCs using a modified Delphi strategy. The discussions resulted in a general MG treatment strategy with specific recommendations for:

- gMG adult patients with specific considerations based on the autoimmune antibodies (AChR + , LRP4 positive (LRP4 +), MuSK positive (MuSK +), or seronegative),
- oMG,
- (Impending and manifesting) myasthenic crisis.

Within these groups, treatment is further stratified according to the level of disease control, in which an add-on and tapering strategy is discussed in case treatment goals are (un)met. In a separate section, comorbidities, drugs aggravating MG symptoms, pregnancy, and vaccination are discussed. Additionally, some perspectives on (near) future MG treatments with high likelihood of market approval and impact on Belgian clinical practice are given.

## Recommendations for treatment

The treatment goals for MG are achievement of minimal symptoms, or improvement in patients whilst minimizing treatment side effects. Currently, there is no curative treatment for MG [20]. MG management aims to restore patients’ muscle strength and well-being through controlling disease activity, monitoring treatment-related adverse events, and individualized supportive measures [6]. A personalized treatment strategy considers thymus pathology, the presence of MG-related antibodies, weakness distribution and severity, patient characteristics, and comorbidities. MG treatment is evaluated at regular intervals based on the patient’s disease classification (Myasthenia Gravis Foundation of America (MGFA) classification (Supplementary Appendix 1) [17]), a clinical evaluation of the patient and an assessment of the impact of the disease on daily life (MG-specific ADL questionnaire (MG-ADL) (Supplementary Appendix 2)) [22]. Due to possible treatment side effects, patients should be periodically monitored.

**Fig. 1:**
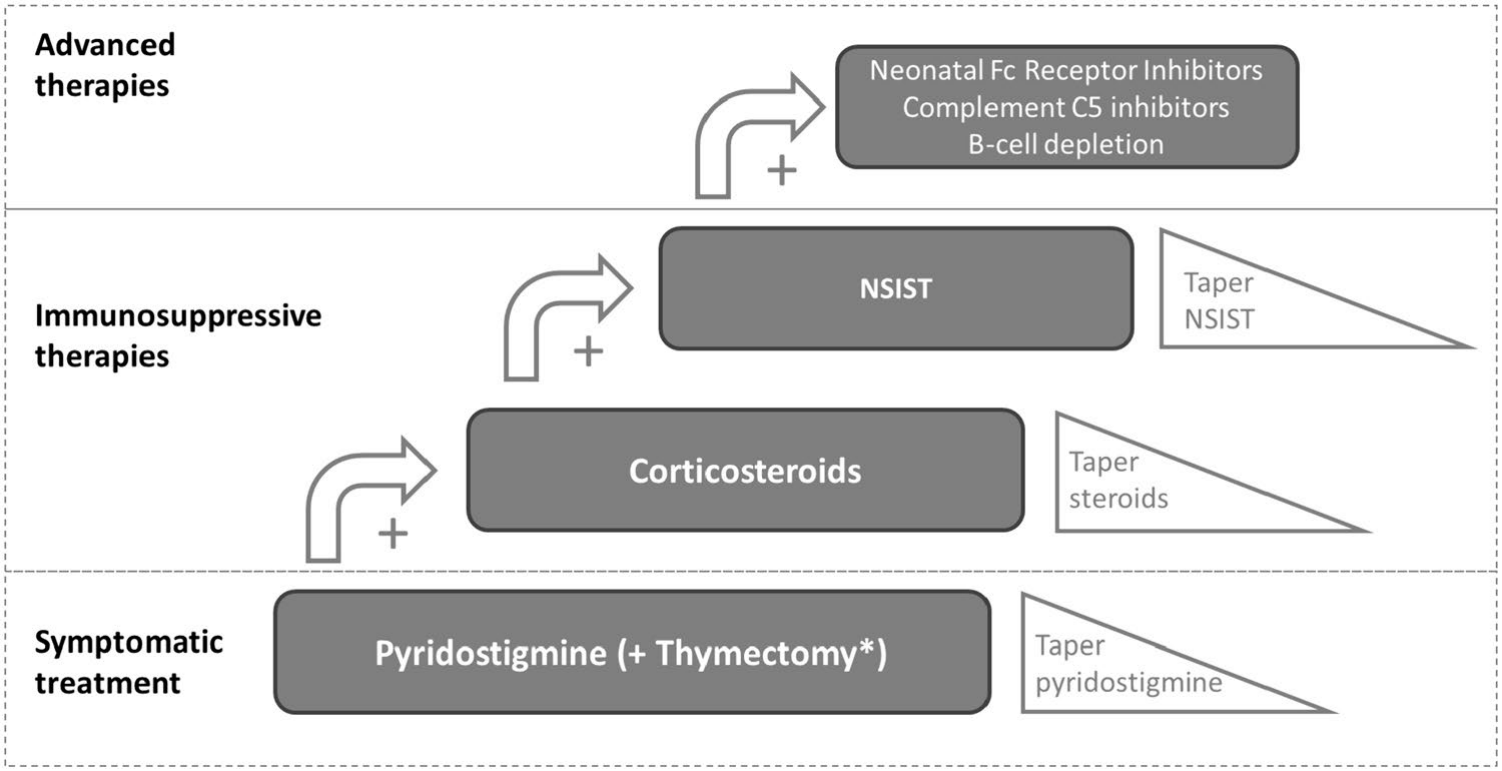
Pharmacological MG treatment strategy including add-on treatments and tapering. *NSIST* non-steroidal immune-suppressive treatment. *Thymectomy should be considered in patients presenting with thymoma or thymic enlargement, and in acetylcholine receptor positive patients between 18 and 50 years without thymoma

If there is insufficient treatment response or the patient experiences too many side effects, another treatment is added or the treatment is switched (Fig. 1). If the patient responds well and is stable for two years on non-steroidal immunosuppressive therapies (NSISTs), treatment can be tapered or even completely removed [17]. Although complete stable remission (CSR) (i.e. the stable absence of symptoms or signs of MG for at least 1 year without treatment during that time [23]) is the goal, which ideally occurs after a temporary treatment course, many patients do not achieve permanent or CSR. These patients experience relapses and/or (slow) disease progression, and are in need of permanent (low-dose) treatment to achieve their best possible outcome [15]. Patients treated inappropriately at time of diagnosis are less likely to achieve CSR [24].

Pharmacological MG treatment typically consists of a combination of thymectomy, symptomatic treatment (acetylcholinesterase inhibitors (AChEIs)), immunosuppressive treatments (corticosteroids and NSISTs), and/or advanced therapies. In case of exacerbations or myasthenic crisis short-term fast-acting treatments such as plasmapheresis and intravenous immunoglobulins (IVIg) can be used. However, in Belgium IVIg is not reimbursed for the indication of MG.

### MG treatment strategy

#### Symptomatic treatment

The oral AChEI pyridostigmine is the first-line treatment [3, 8, 9, 25] (Table 1). Responsiveness to pyridostigmine varies between MG patients in the degree of weakness relief, optimal dosage, and tolerability [26]. Pyridostigmine will restore prolonged muscle strength to normal levels in only a small patient subset. Atropine can be used to counteract the cholinergic side effects of pyridostigmine. Atropine is widely available and effective with a short action onset. Side effects include dry mouth, blurred vision, urinary retention, tachycardia and confusion. These limit its use and warrant careful monitoring. Therefore, atropine is not recommended for this purpose.

**Table 1:** Currently available treatment regimens, action onset time, frequently reported side effects and prevention thereof

Medication	Start dose	Maintenance dose	Action onset	Frequently reported side effects	Prevention of side effects
**Symptomatic treatment**					
Pyridostigmine ! CI in patients with mechanical obstruction of the intestines or urinary tracts!	30 mg; 1–3dd	30–90 mg; 3-6dd (max 720 mg dd and max 120 mg per intake)	30–45 min	Hyperhidrosis, sialorrhea, epiphora, nausea, diarrhoea, polyuria, fasciculations, muscle cramp	Consider cardiac evaluation prior to initiation of pyridostigmine. Reduce dose in case of reduced kidney functioning
**Immunosuppressive treatment**					
Corticosteroids ! CI in patients with ocular herpes simplex !	32–64 mg methylprednisolone dd (40–80 mg prednisolone dd)	32–64 mg methylprednisolone (40–80 mg prednisolone), followed by slow tapering to lowest possible dose	4–8 weeks	Hypertension, hyperglycaemia, weight gain, osteoporosis, skin atrophy, cardiovascular disease, dyslipidaemia, cataract, glaucoma, increased infection risk, irritability, initial worsening of symptoms, steroid myopathy	Blood pressure and glycaemic and lipid control, follow-up and treatment if needed. Bone loss prevention. Slow initiation in patients with bulbar/respiratory complaints. Optimize vaccination status, live (attenuated) vaccinations preferably before starting treatment. Avoid grapefruit (juice)
Azathioprine !Treatment with allopurinol is a CI!	50 mg dd	2–3 mg/kg/dd, by increasing 50 mg every 2–4 weeks	6–12 months	Nausea, diarrhoea, fever, muscle and bone pain, fatigue, rash, hair loss, leukopenia, thrombocytopenia, skin tumours	CBC, liver function, reduce exposure to direct sunlight, use of highly protective sun blockers, TPMT assessment prior to initiation. Optimize vaccination status, live (attenuated) vaccinations preferably before starting treatment
Mycophenolate Mofetil ! Pregnancy is CI!	250–500 mg; 2 dd	Increase weekly with 500 mg until 1000 mg; 2 dd	3–4 months	Infections, leukopenia, anaemia, thrombopenia, hypercholesterolaemia, hyperglycaemia, alkalosis, hyperphosphatemia, confusion, depression, sleep disturbances, anxiety, dizziness, headache, hypertension, diarrhoea, nausea, emesis, constipation	Reduce exposure to direct sunlight, use of highly protective sun blockers, CBC and liver function monitoring. Optimize vaccination status, live (attenuated) vaccinations preferably before starting treatment
Tacrolimus	3 mg dd or 0.1 mg/kg/dd	3 mg dd or 0.1 mg/kg/dd	2–3 months	Nephrotoxicity, lymphoma, skin cancer, anaemia, leukopenia, thrombocytopenia, hyperglycaemia, alkalosis, sleep disturbances, tremor, headache, hepatotoxicity, rash, dyspnoea, hypertension, diarrhoea, nausea, nephrotoxicity	Reduce exposure to direct sunlight, use of highly protective sun blockers, BP and kidney function monitoring. Avoid St John’s wort. Optimize vaccination status, live (attenuated) vaccinations preferably before starting treatment
Cyclosporine	25–100 mg 2dd	3–6 mg/kg/dd in 2 doses (through level < 300 ng/ml in blood)	1–3 months	Nephrotoxicity, tremor, hirsutism, hypertension, diarrhoea, anorexia, nausea, emesis, leukopenia, hyperlipaemia, hepatotoxicity	BP, kidney and liver function monitoring, CBC, reduce exposure to direct sunlight, use of highly protective sun blockers. Avoid St John’s wort. Optimize vaccination status, live (attenuated) vaccinations preferably before starting treatment
**Advanced therapies**					
Rituximab	Refer to NMRC	Multiple initiation and maintenance schemes used	8–16 weeks	Progressive multifocal leukoencephalopathy (PML), infections, infusion reactions, nausea, rash, pruritus, fever, headache, neutropenia, leukopenia, reactivation risk for hepatitis B and tuberculosis	CBC, kidney and liver function monitoring. Optimize vaccination status, live (attenuated) vaccinations preferably before starting treatment. Check hepatitis B and tuberculosis status prior to treatment
Eculizumab	Refer to NMRC 900 mg	900 mg weekly for 4 weeks, 1200 mg at week 5, from then on 1200 mg biweekly	2–12 weeks	Pneumonia, UTI, leukopenia, anaemia, sleeping disturbances, hypertension, dizziness, URTI, diarrhoea, nausea, emesis, joint and muscle pain, fever, infusion reactions	Optimize vaccination status, live (attenuated) vaccinations preferably before starting treatment [27].Obligatory meningococcal vaccination before start
Efgartigimod	Refer to NMRC 10 mg/kg	10 mg/kg, treatment cycles of 4 weeks, with IV infusions once weekly	1–4 weeks	URTI, UTI, myalgia, headache during administration, nasopharyngitis	Optimize vaccination status, live (attenuated) vaccinations preferably before starting treatment

*BP* blood pressure, *CBC* complete blood count, *CI* contra-indication, *dd* per day, *IV* intravenous, *NMRC* neuromuscular reference centre, *TPMT* thiopurine *S*-methyltransferase activity, *URTI* upper respiratory tract infection, *UTI* urinary tract infection

The list of side effects is non-exhaustive. Information in this table is based on Alhaidar et al., 2022; Bubuioc et al. [2]; Farmakidis et al. [27]; Heo et al. [41]; Howard et al. [63]; SmPCs from the respective treatments and the Dutch guidelines on the management of MG produced by Spierziektencentrum Nederland, 2022

#### Immunosuppressive therapies

Most patients with gMG require additional therapy directed at the underlying immune dysregulation at some point in time, if not indefinitely [5, 15, 17, 27]. Glucocorticoids and/or NSISTs are indicated for patients who remain significantly symptomatic under pyridostigmine. Methylprednisolone or equivalent is the first-line immunosuppressive therapy for MG patients due to their relatively rapid effect (Table 1). The dosing regimen and strategy depend on the patient’s characteristics and comorbidities. In gMG patients, starting high-dose steroids can lead to transient worsening of the symptoms. Therefore, slowly increasing the dose initially may be preferred in some (especially bulbar/respiratory affected) patients. Very late onset gMG patients often require lower treatment doses and show less drug resistance [28]. First improvements can be noted from 4 weeks on. Long-term steroid use is associated with side effects [29] (Table 1), and the number and severity of adverse events associated with corticosteroid treatment increases with treatment duration, cumulative dosage, and age [27].

Once an adequate clinical response is obtained, glucocorticoids should be gradually tapered to the lowest dose necessary to maintain disease stability. Slow tapering of glucocorticoids is tried over 9–12 months and depends on the severity of initial symptoms, adverse events, and comorbidities. At lower doses (8 mg methylprednisolone and lower), caution is warranted, and the pace of further tapering should be reduced to prevent disease reactivation. Stopping completely with steroid treatment without combining NSIST treatment, often results in relapse [5].

NSISTs are added in case of insufficient response to glucocorticoids, for corticoid sparing, inability to taper steroids below a reasonably acceptable level without return of symptoms (corticoid-dependence), or intolerance to chronic steroids [12, 16, 30]. NSISTs (azathioprine, mycophenolate mofetil, cyclosporine, tacrolimus) are non-specific, systemic treatments that act via broad immunosuppressive mechanisms [16, 17, 20, 31]. Expert consensus and scientific evidence support the use of azathioprine or mycophenolate mofetil as first choice of NSIST [16]. Choice of treatment depends on, amongst others, tolerability profile and action onset (Table 1). Tacrolimus and cyclosporine can be used as first choice of NSIST in patients hypersensitive to azathioprine/mycophenolate mofetil or when the latter are contraindicated. Considering the delayed response of NSISTs (Table 1), NSIST treatment may also be initiated together with glucocorticoids. Plasmapheresis can be used as bridging therapy until the glucocorticoids or NSISTs start working [6]. Long-term use of NSISTs may be associated with severe adverse events and requires adequate monitoring (Table 1).

#### Advanced therapies

Approximately 15–20% of patients are considered refractory and experience frequent clinical relapse upon tapering their immunotherapy, develop severe side effects or require unacceptably high doses of glucocorticoids despite the concurrent use of NSISTs [32]. Patients in need of advanced treatment should always be referred to an NMRC [16, 30, 32]. Referral to an NMRC can be done in other MG patients as well if deemed necessary. Currently available advanced treatments are B cell depletion therapies (e.g., rituximab), complement C5 inhibitors (e.g., eculizumab), and neonatal Fc receptor inhibitors (FcRn) (e.g., efgartigimod). The choice of advanced treatment depends in part on the MG-related autoimmune antibody.

### Generalized MG

Treatment responsiveness and eligibility for advanced treatment in gMG differs depending on the presence of thymoma, the MG-related autoimmune antibody and age of onset.

#### Presence of thymoma or thymic enlargement

If thymoma or thymic enlargement are detected by imaging, surgical resection of the thymus (thymectomy) is mandatory and should be performed by an experienced team [33]. Depending on the clinical condition, plasma exchange can be given to improve respiratory weakness prior to surgery in patients with dysphagia for solid foods or liquids, or respiratory problems. Incompletely resected thymomas should be further managed after surgery with an interdisciplinary treatment approach (radiotherapy, chemotherapy) based on histological classification [15]. In non-thymomatous hyperplasia, thymectomy is an elective procedure due to the long delay in onset of effect (up to 3 years) and should only be performed when the patient is clinically stable and is deemed safe to undergo a procedure causing postoperative pain and mechanical factors that limit respiratory function [16]. Current data support thymectomy even when performed years after the disease onset [34].

#### AChR + 

**Fig. 2:**
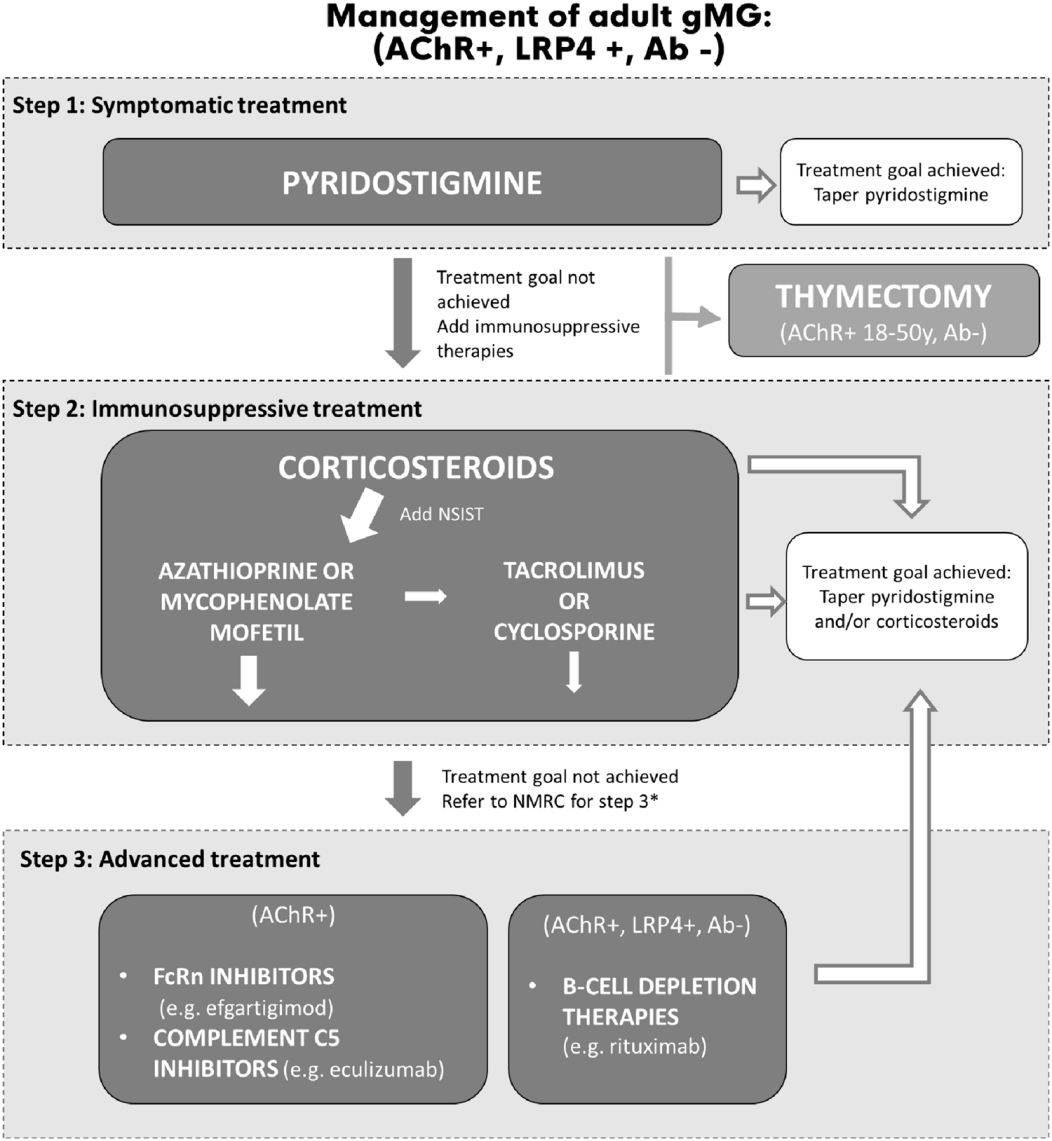
Treatment of generalized adult gMG (AChR + , LPR4 + , Ab −). *Ab* antibody, *AChR* acetylcholine receptor, *FcRn* neonatal Fc receptor, *gMG* generalized myasthenia gravis, *LPR4* low-density lipoprotein-related receptor 4, *NMRC* neuromuscular reference centre, *NSIST* non-steroidal immunosuppressive treatment. *Referral to an NMRC is not required for the prescription of rituximab. Referral to an NMRC may be useful earlier in case of clinical need

The first choice of treatment for adult AChR + gMG patients is pyridostigmine (Fig. 2). AChR + patients have a 90% a priori chance of positive impact of pyridostigmine on disease manifestations [32]. In AChR + patients without thymoma, aged 18–50 years, thymectomy should be considered preferably in the first five years after disease onset to improve clinical outcomes and minimize long-term exposure to pharmacotherapy [6, 16]. The age limit is based on research including patients 18–60 years, yet the results do not support thymectomy in a subgroup of patients aged 50 or older [14, 16].

If symptoms in AChR + gMG patients are not properly controlled with symptomatic treatment and/or immunosuppressive therapies, these patients are eligible for add-on treatment with FcRn inhibitors (e.g., efgartigimod) [30, 32] or complement C5 inhibitors (e.g., eculizumab), [35]. In Belgium, prescribing the latter two drug classes is limited to neurologists attached to an NMRC, and to patients meeting reimbursement criteria. Treatment choice will depend on patient comorbidities, time to action onset, and side effects profile of the drug (Table 1). Comparative data are not yet available.

#### LRP4 + and seronegative Ab gMG

The first choice of treatment in the rare LRP4 + and the more common seronegative gMG patients is pyridostigmine [36] (Fig. 2). In Ab- patients, that are often difficult to treat, thymectomy can be considered if the patient fails to respond adequately to immunotherapy or to avoid intolerable treatment side effects [16]. Clinical evidence does not support thymectomy in LRP4 + gMG patients [16].

Treatment with rituximab should be considered in refractory LRP4 + or Ab- patients, which is reimbursed in Belgium for patients with life-threatening autoimmune diseases. Efgartigimod and eculizumab are not reimbursed in LRP4 + and Ab- gMG patients in February 2024. Clinical studies investigating the effect of advanced therapies, including other FcRn inhibitors, are ongoing in these populations.

#### MuSK + 

**Fig. 3:**
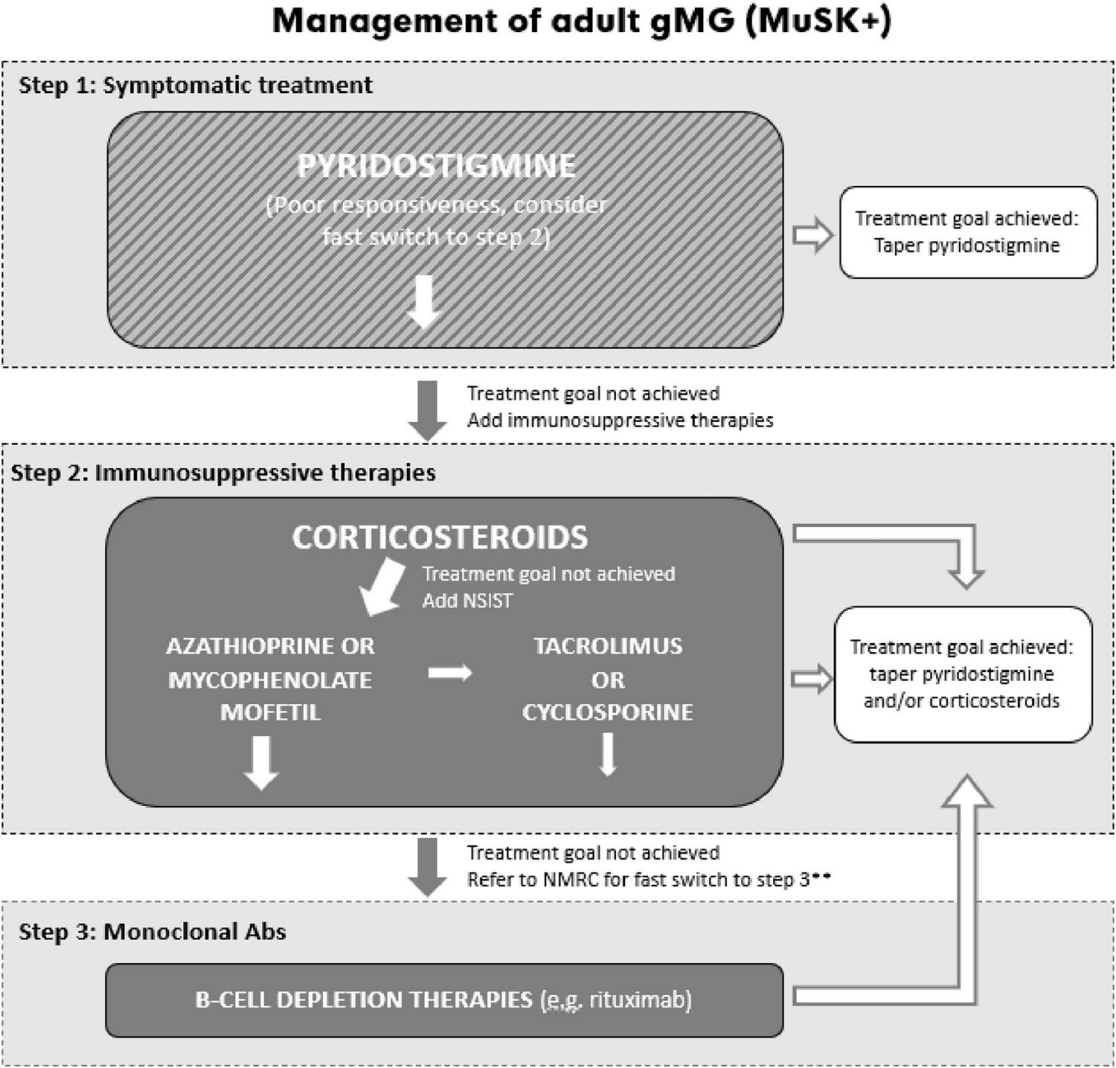
Treatment of adult gMG (MuSK +). *gMG* generalized myasthenia gravis, *NMRC* neuromuscular reference centre, *NSIST* non-steroidal immunosuppressive treatment. *Referral to an NMRC is not required for the prescription of rituximab. Referral to an NMRC may be useful earlier in case of clinical need

Although the responsiveness to pyridostigmine in MuSK + gMG patients is low and frequently induces side effects [15], pyridostigmine is still considered the first choice of treatment [32]. Due to the low responsiveness, adding immunosuppressive treatment should be considered early in the disease course. When at least partially responsive to corticosteroids, NSIST should be added to corticoid treatment. However, if the patient is unresponsive to corticosteroids, B cell depletion (e.g., rituximab) should be considered early on [30, 32] (Fig. 3). Plasmapheresis can be considered as a bridging therapy in patients with severe bulbar symptoms. Thymectomy is not indicated. Clinical studies are ongoing in MuSK + gMG patients investigating the effect of advanced therapies, such as FcRn inhibitors.

### Ocular MG

**Fig. 4:**
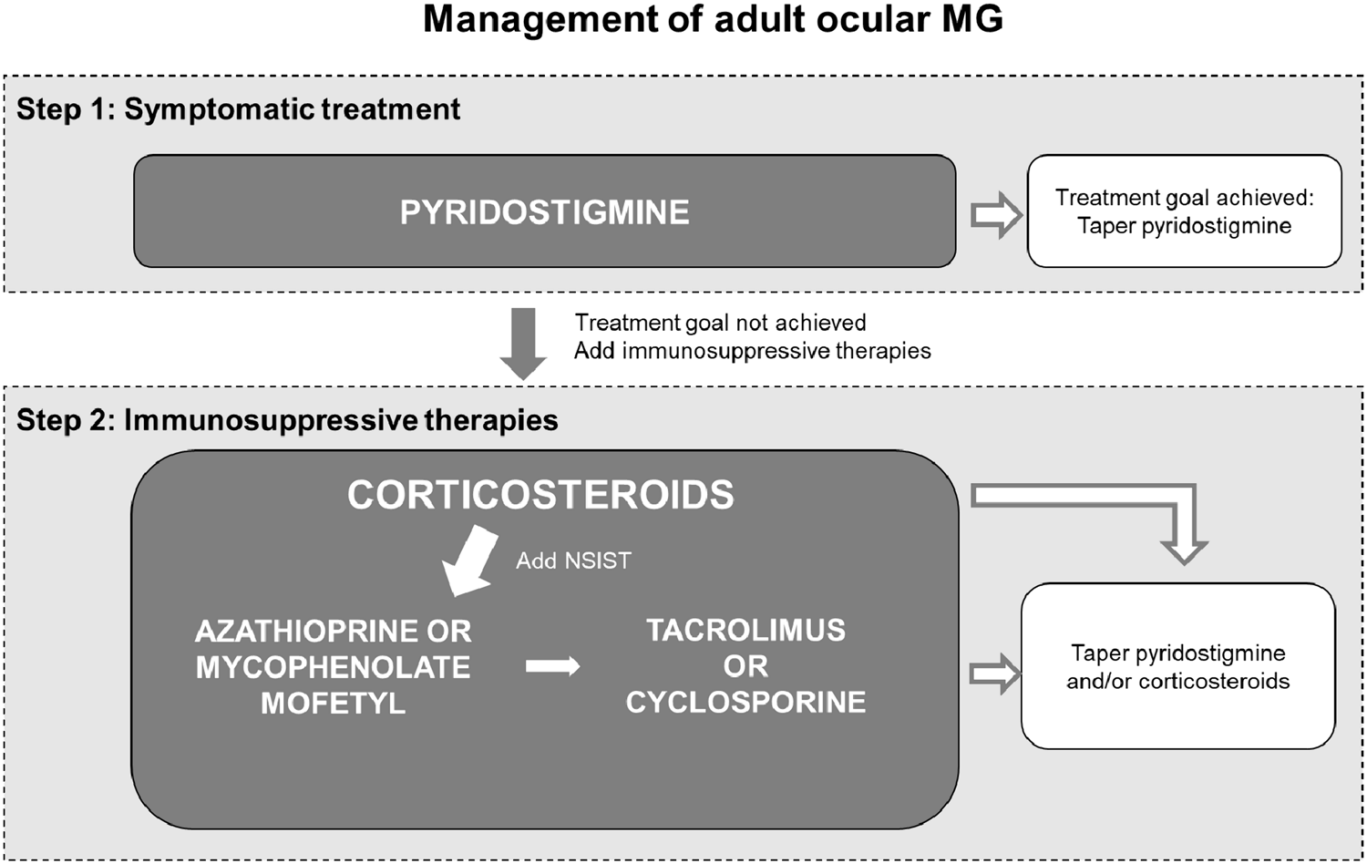
Treatment of ocular MG. *MG* myasthenia gravis, *NSIST* non-steroidal immunosuppressive treatment

Treatment recommendations for oMG are slightly different than those for gMG (Fig. 4). Symptomatic treatment or immunosuppressive treatment is also recommended in oMG patients, but clinically significant responses to pyridostigmine are only observed in half of them [16, 37]. Initiation with corticosteroids should start at low doses [16]. oMG patients are not considered eligible for treatment with monoclonal antibodies as the effectiveness of these treatments has not been properly investigated. However, it must be noted that treatment effects have been observed in patients with gMG with ocular symptoms, which could assume similar effects in oMG. More research in this patient population is required [30] as evidence of early treatment lowering the risk of progression to gMG is under discussion [38–40]. Considering the debilitating impact of diplopia, prism foil or prism glasses can be offered to the patient to reduce its consequences until treatment resorts effect. However, due to the variability of diplopia, prism foil or glasses often do not produce the desired effect.

### Impending and manifesting myasthenic crisis

Some patients experience rapid clinical worsening of MG, not responding to increasing doses of corticosteroids, which may lead to a myasthenic crisis within a short period of time (days to weeks), referred to as an impending myasthenic crisis [27]. The management of an impending and eventually a manifesting myasthenic crisis depends on the pace of deterioration. In an impending myasthenic crisis, plasmapheresis should be initiated [15, 16] as well as high-dose corticosteroids [12], due to the short-lived effect of plasmapheresis [27]. IVIg is a treatment option, but is currently not reimbursed in Belgium. Ideally, efgartigimod or eculizumab is initiated in insufficiently controlled patients, which should resort results within two weeks [41]. However, scientific evidence of their use in myasthenic crisis is currently lacking.

A manifesting myasthenic crisis is defined as severe weakness of the bulbar and respiratory muscles requiring intubation or non-invasive ventilation to maintain airway pressure [15]. MG crises are often induced or accompanied by infections such as pneumonia [42]. A myasthenic crisis is an emergency that should be addressed in an intensive care unit with fast-acting treatments [15, 16, 43]. In Belgium, plasmapheresis is mainly used in crises [15, 16, 43]. Plasmapheresis requires a series of usually five sessions every other day, but has a short-lived effect, requiring the immediate initiation (or increased dosing) of corticosteroids. Obviously, plasmapheresis cannot be combined with monoclonal Ab therapies.

### Other considerations

#### Comorbidities

Comorbidities represent a significant challenge for MG patients and can heavily impact QoL, daily functioning, short-term and long-term outcomes, and mortality [44]. MG patients are at increased risk for concomitant autoimmune disease, especially in early-onset MG. Up to 15% of MG patients have a second autoimmune disease, of which thyroid disease, systemic lupus erythematosus, and rheumatoid arthritis are most common [1, 45]. Non-associated comorbidities, such as diabetes or hypertension, may further hamper treatment, or worsen MG outcome and prognosis, especially in the elderly [13].

#### Drugs that induce or cause deterioration of MG

Certain drugs, such as immune checkpoint inhibitors (used in oncological treatments), penicillamine, interferons or tyrosine kinase inhibitors can elicit an autoimmune reaction against the neuromuscular junction causing de novo MG or an exacerbation in patients with pre-existing MG [16]. MG induced by immune checkpoint inhibitors usually involves bulbar and respiratory muscles and is severe to life-threatening when associated with myositis and/or myocarditis [46]. Early and aggressive treatment with high-dose corticosteroids is advised. However, the decision to stop treatment with immune checkpoint inhibitors should be determined based on the oncologic status and severity of MG [16]. Immune checkpoint inhibitors can worsen the disease in patients with pre-existing MG but are not considered an absolute contra-indication, when the disease is well controlled, and patients are being monitored closely.

Other drugs such as certain antibiotics, antiarrhythmics, anaesthetics and neuromuscular blockers interfere with neuromuscular transmission and can result in an exacerbation or unmasking of MG symptoms [47].

#### Pregnancy

MG is not a contra-indication for pregnancy, yet MG response to pregnancy may vary, from no change in health status to a deterioration in 30–40% of patients, or to an improvement of MG symptoms during the second or third trimester [48, 49]. It is recommended to avoid pregnancy during the first two years following onset of gMG due to the increased risk of myasthenic crisis during this period [49]. Pregnancy planning should be installed well in advance in order to optimize the patient’s clinical status and minimize foetal risks due to medication intake [16, 17, 50] and transplacental passage of MG-causing antibodies. Ideally, a multidisciplinary team including at least an obstetrician, neurologist, anaesthesiologist, and neonatologist should surround the patient during pregnancy, partum, and post-partum. The following considerations are the most important points of attention for MG treatment during pregnancy; more detailed information can be found in specific literature [15, 48–52]:

- Thymectomy should be considered prior to pregnancy or postponed until after pregnancy [16].
- Pyridostigmine and prednisone in low doses are considered safe during pregnancy, however, pyridostigmine may cause premature uterus contractions at the end of pregnancy [16, 17].
- Azathioprine and cyclosporine are considered relatively safe during pregnancy [16, 17], yet during third trimester warrant cautious blood monitoring as medication-induced leukopenia in the mother is a risk factor for neonatal leukopenia [53].
- Mycophenolate mofetil is teratogenic and should not be used during pregnancy [16], with cessation of mycophenolate mofetil at least 6 weeks prior to conception [54].
- Eculizumab treatment during pregnancy does not seem to infer any increased risk for the mothers and their babies. The manufacturer states that eculizumab should only be given during pregnancy if absolutely needed [55, 56].
- Plasmapheresis can be used in case of exacerbations or when intensified therapy is needed.

#### Vaccination

MG patients on immunosuppressive therapies are at increased risk for infection and should be protected by vaccination with influenza, pneumococcal [27] and SARS-Cov-2 vaccines [57, 58]. Ideally, patient vaccination status is optimized before the initiation of immunosuppressive treatment to improve immunogenicity. In patients already on immunosuppressive treatment, the latter should not be interrupted for the administration of inactivated vaccines. The administration of live attenuated vaccines should be avoided as much as possible.

### Future perspectives

**Fig. 5:**
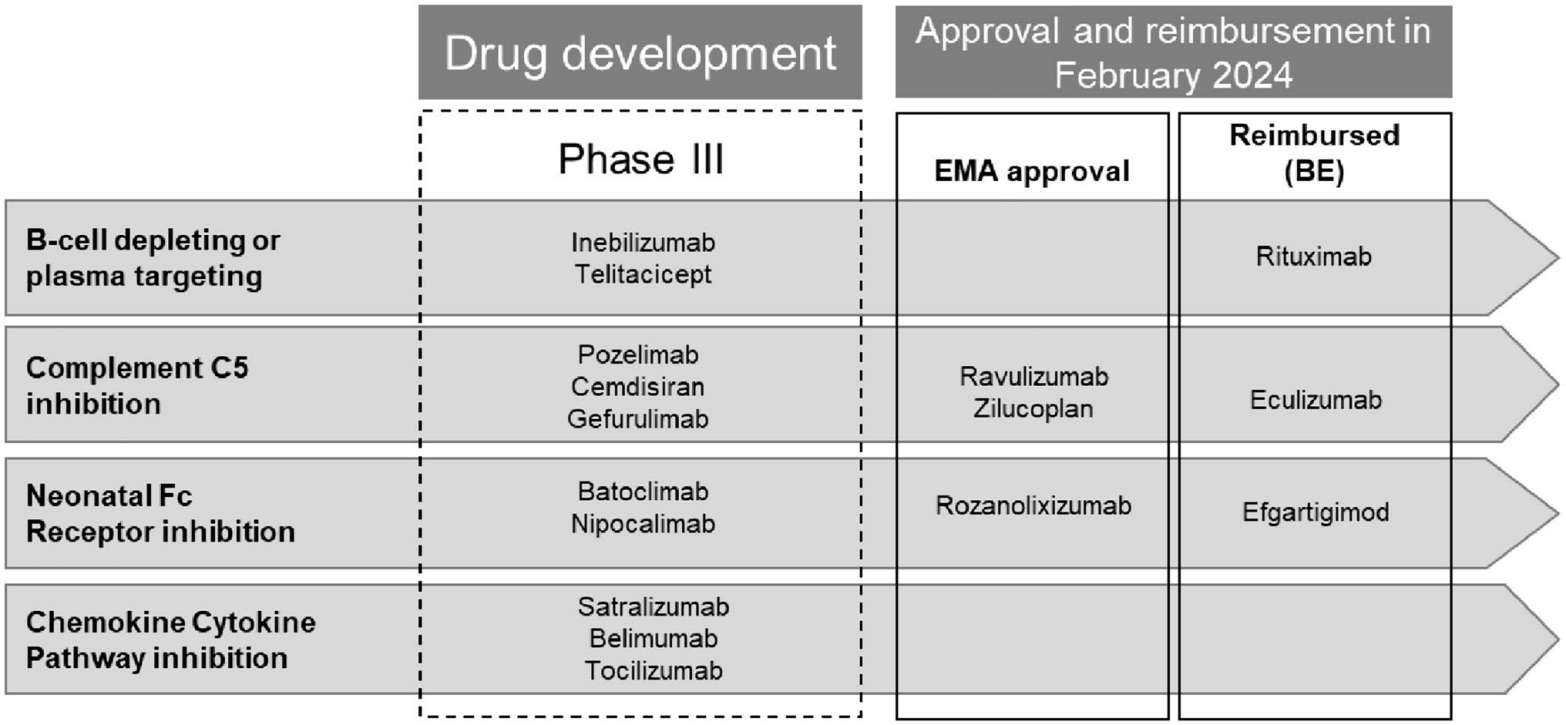
Overview of a selection of new and emerging therapies for myasthenia gravis. Molecules in Phase II development are not included in this overview

Despite the availability of multiple treatment options that help a proportion of patients, many patients still suffer from a high disease and treatment burden with an impact on morbidity, mortality, ADL, productivity and QoL. In addition, an estimated 10–20% of patients with MG are not achieving adequate response or are intolerant to conventional treatment [59]. Side effects and concomitant pathologies are the most important reasons making it difficult to treat some patients. On top, the long delay between treatment initiation and the effective response is a limitation of current treatment options. In recent years, research has identified new, promising treatments, including B-cell depletion or plasma cell targeting treatments [60], complement C5 inhibitors [61–63] and neonatal Fc receptor antagonists [35, 64–66] (Fig. 5).

### Supplementary Information

Below is the link to the electronic supplementary material.

Supplementary file1 (DOCX 23 KB)

Supplementary file2 (DOCX 25 KB)

## Data Availability

Not applicable.
