# Supplementary material for: Recommendations for the management of myasthenia gravis in Belgium
Source: Acta Neurol Belg. 2024 Apr 22;124(4):1371–83. doi: 10.1007/s13760-024-02552-7 (PMC11266451; doi:10.1007/s13760-024-02552-7)
Supplement: Supplementary file 1 — Supplementary file1 (DOCX 23 KB) [file 13760_2024_2552_MOESM1_ESM.docx]

## Recommendations for the management of Myasthenia Gravis in Belgium

**Journal:** Acta Neurologica Belgica

**Authors:** Jan L. De Bleecker, Gauthier Remiche, Alicia Alonso-Jiménez, Vinciane Van Parys, Véronique Bissay, Stéphanie Delstanche, Kristl G. Claeys^.^

**Corresponding author:** Jan de Bleecker (jan.debleecker@ugent.be)

**Appendix 1:** MGFA Classification

| **Class** |  | **Characteristics/Clinical manifestation** |
| --- | --- | --- |
|  |  |  |
| I |  | Any ocular muscle weakness; may have weakness of eye closure. |
|  |  | All other muscle strenght is normal. |
|  |  |  |
| II |  | Mild weakness affecting muscles other than ocular muscles. |
|  |  | May also have ocular muscle weakness of any severity. |
|  | IIa | Predominantly affecting limb, axial muscles, or both. |
|  |  | May also have lesser involvement of oropharyngeal mucles. |
|  | IIb | Predominantly affecting oropharyngeal, respiratory muscles, or both. |
|  |  | May also have lesser or equal involvement of limb, axial muscles, or both. |
|  |  |  |
| III |  | Moderate weakness affecting muscles other than ocular muscles. |
|  |  | May also have ocular muscle weakness of any severity. |
|  | IIIa | Predominantly affecting limb, axial muscles, or both. |
|  |  | May also have lesser involvement of oropharyngeal mucles. |
|  | IIIb | Predominantly affecting oropharyngeal, respiratory muscles, or both. |
|  |  | May also have lesser or equal involvement of limb, axial muscles, or both. |
|  |  |  |
| IV |  | Severe weakness affecting muscles other than ocular muscles. |
|  |  | May also have ocular muscle weakness of any severity. |
|  | Iva | Predominantly affecting limb, axial muscles, or both. |
|  |  | May also have lesser involvement of oropharyngeal mucles. |
|  | Ivb | Predominantly affecting oropharyngeal, respiratory muscles, or both. |
|  |  | May also have lesser or equal involvement of limb, axial muscles, or both. |
|  |  |  |
| V |  | Defined by intubation, with or without mechanical ventilation, except when |
|  |  | employed during routine post-operative management. Using a feeding |
|  |  | tube without intubation places the patient in class Ivb |
| Source: Jaretzki, A.; Barohn, R. J.; Ernstoff, R. M.; Kaminski, H. J.; Keesey, J. C.; Penn, A. S.; Sanders, D. B. (2000). Myasthenia gravis: Recommendations for clinical research standards. Neurology, 55(1), 16–23. | | |
