# Supplementary material for: Recommendations for the management of myasthenia gravis in Belgium
Source: Acta Neurol Belg. 2024 Apr 22;124(4):1371–83. doi: 10.1007/s13760-024-02552-7 (PMC11266451; doi:10.1007/s13760-024-02552-7)
Supplement: Supplementary file 2 — Supplementary file2 (DOCX 25 KB) [file 13760_2024_2552_MOESM2_ESM.docx]

## Recommendations for the management of Myasthenia Gravis in Belgium

**Journal:** Acta Neurologica Belgica

**Authors:** Jan L. De Bleecker, Gauthier Remiche, Alicia Alonso-Jiménez, Vinciane Van Parys, Véronique Bissay, Stéphanie Delstanche, Kristl G. Claeys^.^

**Corresponding author:** Jan de Bleecker (jan.debleecker@ugent.be)

**Appendix 2:** MG ADL

| **Activity** | **Score = 0** | **Score = 1** | **Score = 2** | **Score = 3** | **Your score** |  |
| --- | --- | --- | --- | --- | --- | --- |
|  |  |  |  |  |  |  |
| Talking | Normal | Intermittent slurring or nasal speech | Constant slurring or nasal speech, but can be understood | Difficult to understand speech |  |  |
|  |  |  |  |  |  |  |
|  |  |  |  |  |  |  |
| Chewing | Normal | Fatigue with solid food | Fatigue with soft food | Gastric tube |  |  |
|  |  |  |  |  |  |  |
|  |  |  |  |  |  |  |
| Swallowing | Normal | Rare episode of choking | Frequent choking necessitating changes in diet | Gastric tube |  |  |
|  |  |  |  |  |  |  |
|  |  |  |  |  |  |  |
| Breathing | Normal | Shortness of breath with exertion | Shortness of breath at rest | Ventilator dependence |  |  |
|  |  |  |  |  |  |  |
|  |  |  |  |  |  |  |
| Brushing teeth or hair | Normal | Extra effort, but no rest periods needed | Rest periods needed | Cannot do one of these functions |  |  |
|  |  |  |  |  |  |  |
|  |  |  |  |  |  |  |
| Arising from chair | Normal | Mild, sometimes uses arms | Moderate, always uses arms | Severe, requires assistance |  |  |
|  |  |  |  |  |  |  |
|  |  |  |  |  |  |  |
| Double vision | Normal | Occurs, but not daily | Daily, but not constant | Constant |  |  |
|  |  |  |  |  |  |  |
|  |  |  |  |  |  |  |
| Eyelid droop | Normal | Occurs, but not daily | Daily, but not constant | Constant |  |  |
|  |  |  |  |  |  |  |
| Source: Wolfe GI, Herbelin L, Nations SP, Foster B, Bryan WW, Barohn RJ. Myasthenia gravis activities of daily living profile. Neurology. 1999 Apr 22;52(7):1487-9 | | | | | | |
